# Global Analysis the Potential Medicinal Substances of Shuangxia Decoction and the Process *In Vivo* via Mass Spectrometry Technology

**DOI:** 10.3389/fphar.2021.654807

**Published:** 2021-04-30

**Authors:** Chenning Zhang, Chuanxin Liu, Hao Wu, Jiaqi Wang, Yu Sun, Runhua Liu, Tianyi Li, Xue Yu, Di Geng, Yi-Kun Sun

**Affiliations:** ^1^ School of Chinese Materia Medica, Beijing University of Chinese Medicine, Beijing, China; ^2^ School of Traditional Chinese Medicine, Beijing University of Chinese Medicine, Beijing, China

**Keywords:** Shuangxia decoction, co-decoction, absorbed blood components, absorbed brain components, molecular docking technology

## Abstract

Shuangxia decoction is an effective traditional Chinese medicine formula for treating insomnia. Up to now, there has not been any report about the effective substances. An omics data processing method based on mass spectrometry technology is used to explore the chemical composition changes of Shuangxia decoction, the components absorbed into the blood and brain, and to explore the anti-insomnia mechanism based on molecular docking technology. Forty-nine chemical components in Shuangxia decoction have been identified, and 51 new components generated by co-decoction have been discovered. It was found that 7,404 compounds of Shuangxia decoction were absorbed into the blood. Forty kinds of known compounds were quickly identified, and 15 new compounds generated by co-decoction were also found to be absorbed into the blood. By using UPLC-MS/MS method, it was confirmed that 10 compounds were absorbed into the blood and 9 compounds were absorbed into the brain. Furthermore, it is found that rosmarinic acid is mainly distributed in the hypothalamus and striatum, and caffeic acid is mainly distributed in the hypothalamus, striatum, and hippocampus. Molecular docking results showed rosmarinic acid, danshensu, and HMLA with GABA_A_ receptor have excellent binding characteristics, even surpassing the proligand. Danshensu and HMLA with dopamine D_2_ receptor also showed good binding energy. Our findings will help to further confirm the mechanism of Shuangxia decoction for relieving insomnia, and we also establish a novel data processing method for supplementing the mechanism of the efficacy of other traditional Chinese medicine formula.

## Introduction

In recent decades, medical scientists with ethnic medicines around the world have gradually become enthusiastic about the research of ethnic medicines that imitate the development of chemical synthesis drugs. Most researchers tend to simplify the complex natural medicine formulations, or screen the certain active single compounds from a natural medicine. There is no denying that the research on a single compound is easier, and the interference factors are significantly reduced. The experimental data are easier to be accepted by people of different cultural backgrounds in the world. More importantly, the research results are also easier to commercialize. Interestingly, during the outbreak of the COVID-19 in 2020, traditional Chinese medicine practitioners produced a complex herb prescription based on the classical Chinese medicine bible, named “Qingfei Paidu decoction,” which is composed of 21 traditional Chinese medicines and has shown good clinical effects in China (Sun et al., 2020a). On the contrary, rare single natural medicine or compound has shown good clinical efficacy. Therefore, the traditional use method of ethnic medicine is still worthy of attention. Exploring the connotation of using herbal medicine combination to treat diseases is of great significance to the development of ethnic medicine, which can greatly enhance the confidence of traditional medicine scientists.

The development of mass spectrometry technology is as significant to the research of traditional Chinese medicine as using the most advanced astronomical telescope to explore the unknown planet in black holes. However, up to now, most people use mass spectrometry to identify the components of traditional Chinese medicines which only rely on the phytochemical literature about the extraction, separation, and identification of natural compounds (Lin et al., 2020; Sang et al., 2020; Tian et al., 2020; Xu et al., 2020). This greatly limits the application of mass spectrometry technology, which does not fully reflect the high-throughput performance of mass spectrometry technology. There may be many chemical changes in the co-decoction of different kinds of Chinese medicine. People are paying more attention to the possibility that co-decoction of Chinese medicine can promote or inhibit the dissolution of each other’s compounds (Yang et al., 2017). However, little attention has been paid to the changes in the chemical composition of their co-decoction, especially the production of new substances may contribute to the curative effect of Chinese medicine.

Shuangxia decoction is a traditional Chinese medicine formula, mainly used to treat insomnia and anxiety, which is composed of *Pinellia ternata (Thunb.) Makino* (Banxia, processed with Glycyrrhizae Radix et Rhizoma extract) and *Prunella vulgaris* L. (Xiakucao). It has been recorded with excellent sedative and hypnotic anecdotes in the classics of Chinese medicine. Modern Chinese medicine clinically confirms that this formula has a better effect on alleviating insomnia (Zhou et al., 1993; Liu et al., 2020). Meanwhile, in the early stage, our research team had verified that Shuangxia decoction owns good sedative–hypnotic effects on model animals, such as Drosophila (Xi et al., 2016; Zhang et al., 2016) and mice (Pei et al., 2013; Sun et al., 2020b). However, so far, there is still no research on the material basis of Shuangxia decoction. Therefore, under the existing technical conditions, clarify the chemical components of Shuangxia decoction after co-decoction, identify the drug-derived components in the plasma after administration of Shuangxia decoction, and explore the drug-derived components in target organs (Brain) have obvious significance to explain the effective material of Shuangxia decoction. Mental diseases often have a strong correlation with neurotransmitters (Qiao et al., 2020; Zhang et al., 2020), and most psychiatric drugs work by interfering with the fate of neurotransmitters or neuromodulators (Sturgess et al., 2010), or play some neurotransmitter-like effects (Sollozo-Dupont et al., 2015). For example, the sedative and hypnotic mechanism of benzodiazepines is to promote the ability of the inhibitory neurotransmitter GABA (Hoyer et al., 2020), fluoxetine works by inhibiting the reuptake of 5-HT, thereby treating anxiety or improving insomnia (Li et al., 2020b). Therefore, the mechanism of Shuangxia decoction is most likely to exert anti-insomnia effects by acting on classical inhibitory neuroreceptors. Molecular docking technology is a widely recognized technology for evaluating the interaction between drugs and targets, and the results can predict the potential mechanism of drug action (Li et al., 2020a), and more and more researchers are applying molecular docking technology to traditional Chinese medicine research, which is very suitable for preliminary exploration of the mechanism of action of complex herbs formula (Duan et al., 2020; Liu et al., 2021).

Generally speaking, the ingredients that enter the tissue often possess more, directly related to the activity of the drug. According to international consensus, neurotransmitter or neuromodulator plays an extremely important role in mental illness, and drugs often play the role of endogenous substance supplements or functional antagonists when alleviating mental illness. A large number of literatures also showed that phenolic acid structure compounds often act by stimulating GABA receptors (Johnston et al., 2006), rosmarinic acid and other compounds from Shuangxia decoction have the same free carboxyl end as endogenous GABA, so they may have similar pharmacophores. Endogenous dopamine has a typical catechol mother nucleus. Rosmarinic acid, ferulic acid, and other series of compounds also have catechol mother nucleus, so dopamine receptors were also selected for molecular docking analysis. Therefore, classical neurotransmitter-driven sedative and hypnotic receptors such as GABA receptors and dopamine receptors are always selected for molecular docking.

In order to confirm the medicinal material of Shuangxia decoction, an omics-based data processing method was used to analyze the chemical composition changes of Shuangxia decoction after co-decoction with UPLC-QE-Orbitrap-MS; MS information of new generated compounds is provided. Meanwhile, the known chemical compositions in Shuangxia decoction were identified based on the literatures of *Pinellia ternata (Thunb.) Makino* and *Prunella vulgaris* L. A global analysis of all absorbed blood components from Shuangxia decoction is also objectively presented. A high-sensitivity and credibility UPLC-MS/MS method was used to disclose the chemical components of Shuangxia decoction absorbed into different brain regions. Finally, molecular docking technology was used to evaluate the binding capacity of the components absorbed into the brain and the classic targets related to neurotransmitters, which may have preliminarily revealed the mechanism of Shuangxia decoction in treating insomnia.

## Materials and Methods

### Chemicals and Reagents

*Pinellia ternata (Thunb.) Makino* (Batch number: 190101) was purchased from Jiangxi Bairen Chinese herbal medicine Co., Ltd (Zhangshu, Jiangxi, China). *Prunella vulgaris* L. (Batch number: 20180901) was obtained by Hubei Shennong herbal Chinese herbal pieces Co., Ltd. (Shiyan, Hubei, China). They were identified by Professor Sun Yikun (Beijing University of Chinese Medicine, Beijing, China). The following agents were involved in the current work: acetonitrile, formic acid, and methanol, all three reagents (LC-MS grade) were purchased from Thermo Fisher Scientific (China) Co., Ltd. Ultrapure water was prepared by a Millipore Alpha-Q water purification system (Millipore, Bedford, United States). All standard reference compounds such as citric acid (Lot: Y27JTY18468), danshensu (Lot: G24J10L91371), 3,4-dihydroxybenzoic acid (Lot: 110810-200506), protocatechualdehyde (Lot: Z30M6L1), 4-hydroxy-3-methyloxyphenyl lactic acid (HMLA) (Lot: Z12011H127185), caffeic acid (Lot: W160108100366), ferulic acid (Lot: Z19032928), salviaflaside (Lot: 20080729), rosmarinic acid (Lot: Y06A9K67402), liquiritigenin (Lot: PS0104481BD02B026), and glycyrrhizic acid (Lot: Z2018X40265) were purchased from Shanghai Yuanye Biological Co., Ltd., and the purity is greater than 98%.

### Preparation of Shuangxia Decoction Extract

*Pinellia ternata (Thunb.) Makino* and *Prunella vulgaris* L. were weighed from the same batch of samples. 15 g *Pinellia ternata (Thunb.) Makino* and 15 g *Prunella vulgaris* L. are decocted with ten times water (w/v) separately. Meanwhile, other 15 g *Pinellia ternata (Thunb.) Makino* and 15 g *Prunella vulgaris* L. are co-decocted with the same approach, and the above sample is used for chemical composition analysis. Sample processing is as follows: the decoction liquid was centrifuged for 10 min with 12000 r/min, and diluted to 5 times, take 5 uL of diluent for mass spectrometry analysis. In addition, five times equal amounts of *Pinellia ternata (Thunb.) Makino* and *Prunella vulgaris* L. are weighed and co-decocted, which used for preparing freeze-dried powder for animal experiments. All the above decoctions are dealt with 2 h each time, a total of two times, combined with the decoction liquid.

### UPLC-QE-Orbitrap-MS Instruments and Conditions

UPLC-QE-Orbitrap-MS separation was achieved on a Waters HSS T3 UPLC C18 column (1.7 µm, 2.1 × 150 mm, Milford, MA, United States) hyphenated with an online filter. The column was eluted with a gradient of 0.1% formic acid aqueous solution (A) and acetonitrile (B) at a flow rate of 0.3 ml/min and a temperature of 40°C: 0–2 min: 5% B; 2–17 min: 5–98% B; 17–19 min: 98–98% B; 19–23 min: 98–5% B; and 23–25 min: 5% B. The injection volume was 5 μL. Data were acquired, and statistics were calculated by Xcalibur software (Thermo Fisher Scientific Corp, New York, MA, United States).

The ion source is an electrospray ionization source (ESI), and positive and negative ions are alternately scanned; the scan mode is full scan/data-dependent two-stage scan (full scan/ddMS^2^), the scan range is 100–1500 Da, and the capillary temperature is 350°C. The spray voltage in the negative mode is 3800 V, the spray voltage in the positive mode is 3200 V, the sheath gas is 35 arb, and the auxiliary gas is 15 arb. MS2 uses low, medium, and high collision energy. The positive/negative ion mode is 30, 40, and 50 V. Resolution of primary mass spectrometry full scan is 70,000 FWHM (full width at half maximum), and resolution of secondary mass spectrometry is MS/MS17500 FWHM.

### UPLC-MS/MS Instruments and Conditions

The UPLC-MS/MS system consisted of an ACQUITY I-Class Plus UPLC system and a XEVO TQS-micro triple quadrupole tandem mass spectrometer (Waters Corp, Milford, MA, United States) equipped with an electrospray ionization (ESI) source. Chromatographic separation was conducted using an UPLC HSS T3 C18 column (2.1 × 50 mm, 1.8 µm; Waters Corp, Milford, MA, United States) at 40°C. Data were acquired and statistics were calculated by Masslynx 4.2 version. Solvent A (0.1% formic acid in ultrapure purity water) and solvent B (acetonitrile) were used as mobile phases. The gradient elution program is as follows: 0–1 min, 5–5% B; 1–9 min, 5–50% B; 9–12 min, 50–95% B, 12–13 min, 95% B; 13–14 min, 95–5% B; and 14–15 min, 5% B. The UPLC auto sampler remained at 10°C. All analytes were quantified using multiple reactions monitoring (MRM) detection in negative ion mode. The transitions, retention time, dwell time, cone voltage, and collision energy parameters are listed in Table 1. Other main working parameters were as follows: capillary voltage 0.5 kV, desolvation temperature 500°C, source temperature 150°C, cone gas (nitrogen) flow 50 L/h, and desolation gas (nitrogen) flow 1,000 L/h.

**TABLE 1:** MS/MS transitions and parameters for the detection of the 10 standards.

Analyte	Retention time	Parent (m/z)	Daughter (m/z)	Dwell (s)	Cone (V)	Collision (V)
Danshensu	1.2	196.8	134.8	0.025	22	16
3,4-dihydroxybenzoic acid	1.37	152.8	108.8	0.025	32	24
Protocatechualdehyde	2.12	136.8	107.9	0.025	36	20
HMLA	2.49	210.9	133.8	0.025	42	16
Caffeic acid	2.76	178.8	134.8	0.025	2	16
Ferulic acid	2.79	192.8	133.8	0.025	4	16
Salviaflaside	4.24	521.1	359.0	0.025	54	14
Rosmarinic acid	4.74	359.0	160.9	0.025	2	14
Liquiritigenin	5.34	254.9	118.8	0.025	46	26
Glycyrrhizic acid	7.67	821.5	351.0	0.025	96	30

### Animals and Treatments

The animal experiment protocol was approved by the Animal Care and Use Committee of Beijing University of Chinese Medicine. Six male Wistar rats (220 ± 20 g, SPF grade) were provided from Sibei Fu (Beijing, China) Experimental Animals Technology Co., Ltd. with the license number “SCXK (Jing) 2016-0002.” All rats were housed in the experimental animal center of Beijing University of Chinese Medicine (Beijing, China) at a temperature and humidity of 23 ± 2°C and 60 ± 5%, respectively. Shuangxia decoction freeze-dried powder was dissolved in purified water and orally administrated to the rats at a dose of 3.5 g/kg (1.5 ml/100 g) crude herbal medicine for single dose per day for a week, and the dose is determined according to the clinical dose and the animal experiment results reported by our research team (Pei et al., 2013; Sun et al., 2020a). The content of danshensu (4.27 mg/g), 3,4-dihydroxybenzoic acid (0.42 mg/g), protocatechualdehyde (0.54 mg/g), caffeic acid (2.69 mg/g), salviaflaside (2.17 mg/g), rosmarinic acid (1.62 mg/g), liquiritigenin (0.3 mg/g), and glycyrrhizic acid (1.58 mg/g) from Shuangxia decoction freeze-dried powder was determined with UPLC-MS/MS.

### Biological Sample Preparation

After one week of continuous administration, 1 h after the last dose, all rats were anesthetized with ether, and blood was taken from the abdominal aorta, and the whole blood was centrifuged at 4,000 rpm for 10 min at 4°C, and the plasma was taken for reserve. Then, the rats were perfused with ice saline, remove brain tissue from rat head, separate the hippocampus, cortex, hypothalamus, striatum, medulla oblongata, and cerebellum on the ice, and store at −80°C for reserve. Take 50 µL of plasma samples or brain tissue samples (1 g/3 ml; brain tissue/physiological saline), add 25 µL of 12% dilute hydrochloric acid, vortex for 30 s, and then, add 10 times ethyl acetate, vortex for 5 min, centrifuge to take the supernatant, repeat once, and combine the supernatant. Dry with nitrogen at 37°C. Finally, 50 µL water was added to reconstitute, centrifuge at 12,000 rpm for 10 min, and take 5 µL sample for analysis.

### Molecular Docking

The structure files of compounds absorbed into brain regions were downloaded from the Chemical Book database (https://www.chemicalbook.com/), using these structures as docked small molecule ligands. Use the Maestro 11.8 software to optimize the structure of the obtained protein through the Protein Preparation Wizard, that is, hydrogenation and distribution of its protonation state and formal charge, delete water molecules with less than 3 hydrogen bonds, and use OPLS_2005 force field to minimize the energy of protein structure in converge heavy atoms to RMSD less than 0.3 Å; Ligprep panel is used to optimize the structure of small molecules of ligands in molecular docking, which mainly defines whether to produce protonated states or stereoisomers. Each small molecule generates no more than 32 structures. Use default parameters to add force fields to small molecule ligands; use the original configuration in the protein structure, the site where the body is located is the basis to define the binding pocket of the protein, use the extra precision (XP) mode to dock all small ligand molecules to the target protein; and score the docking results and compare their respective docking result scores. Generally, the conformation of the compound molecule and the receptor is stable, when the score is small. Before docking the selected ligand, we extract the original ligand of the protein, then dock it back to its binding pocket according to the set parameters, and calculate the root mean square deviation (RMSD) of the conformation after docking and the structure of the original ligand. It is generally believed that when the RMSD ≤ 2 Å, this set of parameters can better reproduce the binding mode of the ligand and the receptor, and the docking method is considered feasible. The interaction between the receptor and the ligand for the two compounds with the best results was plotted for each protein docking. The binding energy ≤ 0 kJ mol^−1^ indicates that the ligand and receptor can bind spontaneously, and the binding energy ≤ -5.0 kJ mol^−1^ proves that the molecule has a good docking with the target (Wu et al., 2020).

### Data Processing and Analysis

The information of high-resolution mass spectrometry to identify the compounds in *Pinellia ternata (Thunb.) Makino* and *Prunella vulgaris* L. was collected from literatures (Liang et al., 2013; Yang et al., 2016; Zhai et al., 2019), respectively. Thus, comparing the precursor ions and fragments to identify the known compounds in Shuangxia decoction, Compound Discoverer 3.1.1.12 is used for screening and identification of other compounds. Analysis Base File Converter and MS-DIAL ver. 4.38 is used for the preprocessing of high-resolution mass spectrometry data, such as the decoction of medicinal materials and rat plasma samples after administration. Retention time and m/z value are used to define a unique compound. Jvenn (http://jvenn.toulouse.inra.fr/app/example.html) is applied for rapid analysis of common compounds or unique compounds in different decoction samples and biological samples, and manually extract the mass spectrometry data and analyze the jvenn results to verify the reliability. Retention time, m/z value, and compound normalized value are used to make bubble chart via Office 2019 and Origin 2021.

## Results

### Differences in Chemical Composition Between Individual Decoction and Co-decoction

**FIGURE 1:**
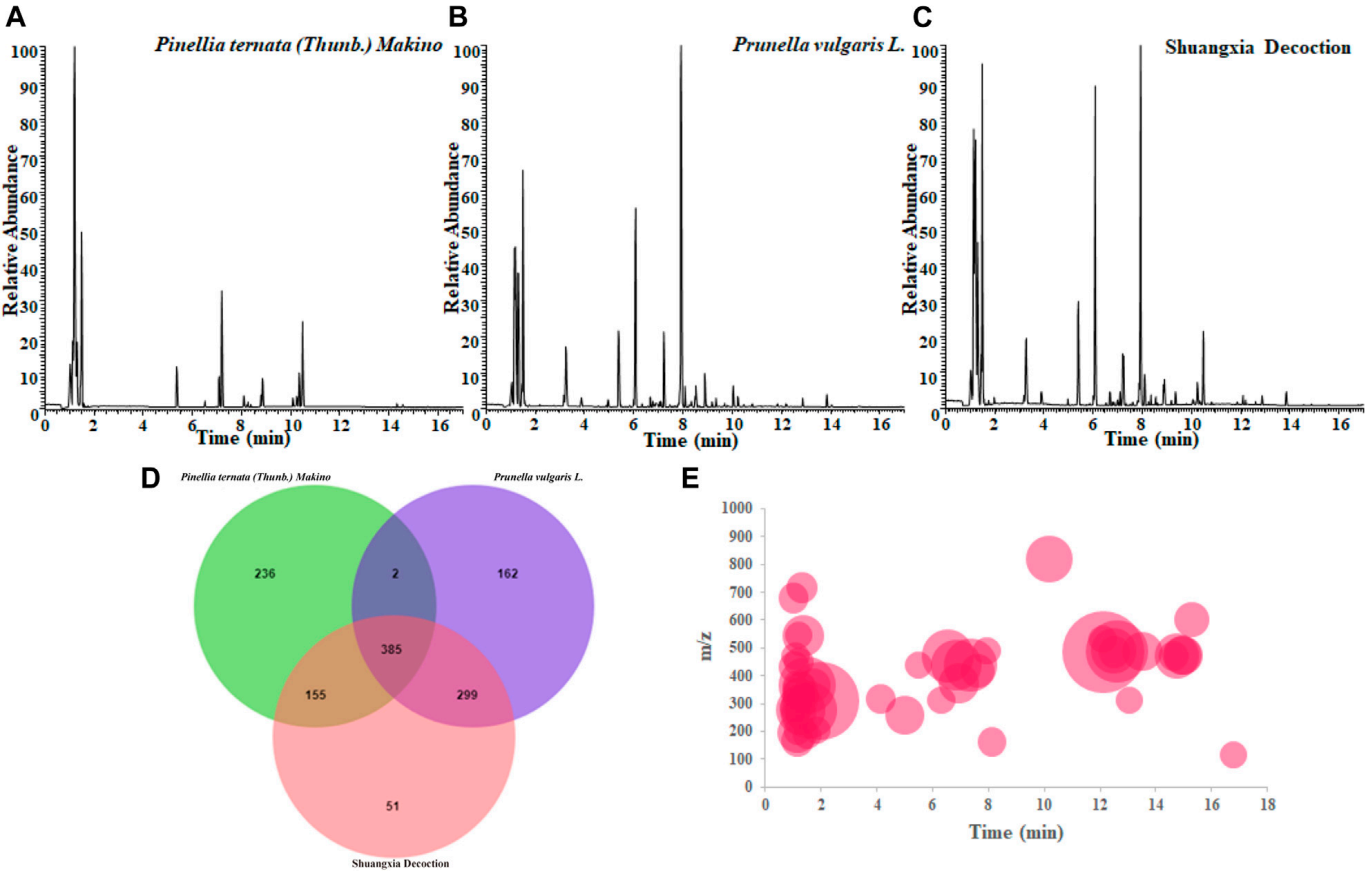
BPI chromatogram of *Pinellia ternata (Thunb.) Makino* **(A)** and *Prunella vulgaris* L. **(B)** individual decoction and the co-decoction (Shuangxia decoction) **(C)**; the main common compounds and unique compounds of the three decoction liquids were analyzed via jvenn website **(D)**; distribution trend of MS information of new generated compounds after co-decoction **(E)**. The chromatographic retention time and m/z value of each new compound are presented on the coordinate axis, and the relative normalized value is expressed by the size of the bubble.

As shown in Figure 1, Figures 1A–C showed the BPI diagrams of *Pinellia ternata (Thunb.) Makino*, *Prunella vulgaris* L., and Shuangxia decoction (SXD), after decoction under the negative ion mode, respectively. Meanwhile, the three samples were also analyzed in the positive ion mode, but almost no chromatographic peaks were visible, so the analysis was abandoned. In most of the literature, researchers are more inclined to identify the chromatograms with higher intensity, but the substances hidden under the chromatographic peaks visible to the naked eye are often ignored. High-resolution mass spectrometry has extremely high resolution, which enables high-throughput analysis capabilities; thus, it may be more meaningful to identify the trace components.

In this study, we normalize all the acquired mass spectrometry data with MSDIAL tools and other online database as metabolomics data processing method, and using retention time and m/z value to simultaneously lock one compound, and perform chromatographic peak extraction verification for possible false positive results, such as with similar retention time or similar m/z ratio, the results prove that the compound obtained by this novel method has absolute specificity, and then intersecting the three sets of data, furthermore, eliminate the values with a small contribution, such as those with a normalized value less than e^5^, then perform statistical analysis. As shown in Figure 1D, interestingly, in the co-decocted Shuangxia decoction, we unexpectedly discovered that 51 compounds did not exist in the decocted alone. In other words, when the two herbal medicines are co-decocted, the compounds contained in them may undergo chemical reactions and generate new compounds. A more direct distribution information of the new generated compounds on the BPI chromatogram is shown in Figure 1E.

The 49 main compounds in Shuangxia decoction were predicted or identified through literature comparison and standard compound data comparison, and the results are shown in Table 2. A variety of components (compounds) from *Pinellia ternata (Thunb.) Makino*, *Prunella vulgaris* L and even the compounds from Glycyrrhizae Radix et Rhizoma have been identified. Of course, some compounds from unidentified sources have been identified. The mass spectrum information of 51 new generated compounds is also presented in Table 3. Due to lack of sufficient structural identification information, these compounds cannot be accurately identified. According to the size of the bubble chart, we can know that compounds such as m/z485.3281, m/z487.3421, and m/z471.0074 have a large contribution and may have potential activity.

**TABLE 2:** Main compound identification of Shuangxia decoction in the negative ion mode relies on the main visible chromatographic peak.

Peak No	tR/min (ion mode)	Molecular Formula	PPM	[M–H]^-^		MS/MS ions	Presumption	Source
				Measured value	Theoretical value			
1	1.55 (−)	C6H8O7	−1.15	191.01884	191.01862	191.0191, 146.93767, 129.01826, 111.00761, 102.94759, 87.00752	Citric acid	PL
2	3.52 (−)	C8H8O4	−0.87	167.03403	167.03389	123.04395, 137.02327, 121.02818, 109.02829	Vanillic acid	PM
3	3.69 (−)	C9H10O5	−1.07	197.04466	197.04445	179.03409, 135.04399, 123.04397, 181.63736	Danshensu	PL
4	4.32 (−)	C7H6O4	−0.13	153.01825	153.01823	153.01846, 142.94557, 114.95053, 109.02839	3,4-dihydroxybenzoic acid	PL
5	4.63 (−)	C13H16O9	−3.34	315.07211	315.07106	271.09769, 153.01830, 123.04393, 109.02822	5-(β-D-glucopyranosyloxy)-2-hydroxybenzoic acid	Unknown
6	4.9 (−)	C10H8O6	−2.58	223.02429	223.02371	179.03403, 161.03900, 151.03900, 135.04399	Herbaric acid	Unknown
7	5.02 (−)	C11H12O7	−3.30	255.05077	255.04993	211.06053, 193.04985, 149.05971, 137.05966	Piscidic acid	Unknown
8	5.13 (−)	C7H8O2	0.86	123.04395	123.04406	95.04893, 93.03328, 81.03320	2,6-dimethyl-4H-pyran-4-one	Unknown
9	5.2 (−)	C9H10O4	−1.13	181.04974	181.04954	163.03905, 137.02330, 135.04399, 119.04899	Homovanillic acid	PM
10	5.24 (−)	C11H9NO2	1.41	188.07034	188.07061	160.07530, 146.05988, 118.06522	1-acetyl-1h-indole-3-carbaldehyde	Unknown
11	5.29 (−)	C20H18O10	−2.37	417.08261	417.08162	329.10272, 285.11304, 267.10257, 135.04404	Juglanin	Unknown
12	5.59 (−)	C7H6O3	1.02	137.02318	137.02332	137.02328, 109.02833, 93.03327, 81.03313	Protocatechualdehyde	PL
13	5.83 (−)	C17H16O8	−2.38	347.07697	347.07614	303.08572, 285.07828, 259.09601, 215.10712	2.2'-[(6-oxo-7.8,9,10-tetrahydro-6h-benzo [c]chromene-1,3-diyl) bis(oxy)]diacetic acid	Unknown
14	6.14 (−)	C9H6O4	−1.44	177.01849	177.01824	135.04395, 133.02838, 105.03338	Esculetin	PL
15	6.19 (−)	C9H8O4	−0.39	179.03395	179.03388	179.07034, 151.07538, 135.04405, 103.91883	Caffeic acid	PL
16	6.5 (−)	C26H28O14	−1.95	563.14063	563.13953	503.11469, 443.10107, 383.078828, 353.06631,	Apiin	PM
17	6.66 (−)	C18H14O9	−2.23	373.05624	373.05541	327.05099, 267.06601, 239.07123	2-(3,4-dihydroxyphenyl)-3,5-dihydroxy-7-methoxy-4-oxo-4H-chromen-8-yl acetate	Unknown
18	6.73 (−)	C17H16O7	−2.69	331.08212	331.08123	283.05994, 255.06682, 197.04469, 179.03406, 151.03899	2-(3,5-dimethoxyphenyl)-5,6,7-trihydroxy-2,3-dihydrochromen-4-one	Unknown
19	6.82 (−)	C15H10O6	−3.98	285.04050	285.03936	255.02980, 241.05019, 211.03934	Kaempferol	PL
20	6.9 (−)	C15H10O4	−3.18	253.05034	253.04954	237.05569, 209.06030, 181.06497	Daidzein	PM
21	6.9 (−)	C15H10O4	−3.18	253.05034	253.04954	237.05556, 225.05478, 209.06038, 181.06500	Chrysophanic acid	PM
22	6.92 (−)	C27H30O16	−2.21	609.14636	609.14501	511.08969, 271.02466, 301.03397, 151.00250	Rutin	PL
23	6.99 (−)	C18H14O9	−1.75	373.05606	373.05541	329.06647, 311.05618, 283.06094, 267.06613	3-(2,4-dimethoxyphenoxy)-5,7-dihydroxy-4-oxo-4H-chromene-2-carboxylic acid	Unknown
24	7.06 (−)	C9H8O3	−0.36	163.03903	163.03897	119.04900, 103.91891	p-coumaric acid	PL
25	7.11 (−)	C21H20O12	−2.82	463.08841	463.08710	301.03375, 271.02457, 255.02937, 243.02777	Hyperoside	PL
26	7.13 (−)	C26H30O13	−1.24	549.16095	549.16027	429.10483, 373.67609, 255.06601, 135.00764	Liguiritigenin-7-O-d-apiosyl-4′-O-D-glucoside	GRER
27	7.24 (−)	C21H22O9	−1.90	417.11880	417.11801	343.08255, 255.06781, 147.02872, 135.04362	Liquiritin (Sh)	GRER
28	7.27 (−)	C24H26O13	−0.71	521.12933	521.12896	521.13025, 359.10226, 329.13928, 197.04474, 179.03415, 161.02342	Salviaflaside	PL
29	7.48 (−)	C27H22O12	−1.76	537.10370	537.10275	493.11588, 331.08228, 311.05542, 161.02335	Lithospermic acid	Unknown
30	7.53 (−)	C17H12O6	−3.46	311.05609	311.05501	269.08154, 159.04414, 147.02885, 109.02825	4-(3-hydroxy-6-methoxy-4-oxo-4H-chromen-2-yl) benzoic acid	Unknown
31	7.83 (−)	C18H12O8	−2.27	355.04565	355.04484	311.05591, 267.06598, 239.07066, 221.06064	2.2′-(1,8-dihydroxy-9,10-dioxo-9,10-dihydroanthracene-2,7-diyl) diacetic acid	Unknown
32	7.87 (−)	C14H24O6	−3.01	287.14978	287.14891	269.13937, 251.12854, 227.12833, 209.11768	[5-hydroxy-3-(hydroxymethyl)pentyl] hydrogen cyclohexane-1,2-dicarboxylate	Unknown
33	7.96 (−)	C18H16O8	−1.03	359.07651	359.07614	359.20828, 197.04474, 179.03416, 161.02341, 135.04396	Rosmarinic acid	PL
34	7.96 (−)	C9H6O3	1.18	161.02313	161.02332	133.02832, 123.94485, 103.91898	7-hydroxycoumarin	PL
35	8.14 (−)	C9H16O4	−0.77	187.09663	187.09649	169.08612, 149.00063, 143.10666, 125.09598	Nonanedioic acid	PM
36	8.28 (−)	C21H22O9	−2.35	417.11899	417.11801	297.07666, 255.06596, 239.07158, 135.00763	Isoliquiritin	GRER
37	8.37 (−)	C27H20O12	−0.91	535.08759	535.08710	355.04581, 311.05591, 267.06601, 239.07106	Sagecoumarin	Unknown
38	8.58 (−)	C18H16O7	−1.98	343.08191	343.08123	197.04475, 179.03413, 161.02341, 145.02841	Eupatorin	Unknown
39	8.89 (−)	C15H12O4	−2.23	255.06575	255.06518	255.06599, 153.01825, 135.0076, 119.049	Liquiritigenin	GRER
40	9.5 (−)	C26H20O10	−1.40	491.09796	491.09727	311.05591, 267.06610, 239.07115, 135.04399	Salvianolic acid C	Unknown
41	9.83 (−)	C18H32O5	−2.11	327.21729	327.21660	291.19641, 229.14410, 211.13338, 171.10168	Seimatopolide A	Unknown
42	10.13 (−)	C42H62O17	0.07	837.39050	837.39056	727.88745, 493.80930, 351.05685, 289.05618	Licoricesaponin G2	PM
43	10.38 (−)	C15H12O4	−2.47	255.06581	255.06518	255.06599, 153.01825, 135.0076, 119.04901	Isoliquiritigenin	GRER
44	10.52 (−)	C42H62O16	0.02	821.39539	821.39541	821.39679, 736.5127, 587.99011, 351.05655, 193.03452, 113.02319	Glycyrrhizic acid	PL
45	10.86 (−)	C36H56O11	−1.12	663.37463	663.37389	587.36243, 487.34280, 439.32211, 113.02319	Phytolaccoside B	Unknown
46	11.39 (−)	C13H24O4	−2.44	243.15968	243.15909	225.14903, 181.15903,	Tridecanedioic acid	Unknown
47	12.12 (−)	C30H46O5	−1.11	485.32669	485.32615	465.30151, 301.21738, 169.08649, 83.04895	Epiceanothic acid	Unknown
48	12.91 (−)	C40H62O13	−0.70	749.41119	749.41067	599.35352, 441.33658, 393.31677, 113.02313	Bidentatoside II	Unknown
49	13.89 (−)	C35H54O9	−0.49	617.36871	617.36841	599.35931, 485.32184, 441.33887, 113.02317	Cimiaceroside A	Unknown

PM, *Pinellia ternata (Thunb.) Makino*; PL, *Prunella vulgaris* L.; GRER, Glycyrrhizae Radix et Rhizoma.

**TABLE 3:** MS information of potential new generated compounds from Shuangxia decoction and co-decoction compared with *Pinellia ternata (Thunb.) Makino* and *Prunella vulgaris* L. decoction, separately.

Peak no	tR/min	[M–H]^−^	MS/MS ions
New-1	1.03	678.81299	669.91, 589.45, 304.91, 288.93, 174.95
New-2	1.05	430.80762	400.79, 374.81, 364.83, 272.85
New-3	1.08	264.87399	198.92, 180.91, 152.91, 147.26
New-4	1.10	470.76233	288.93, 174.95, 158.97, 146.96
New-5	1.11	286.85382	258.86, 230.87, 170.88, 156.89
New-6	1.15	164.83542	136.93, 120.94, 108.93, 96.95
New-7	1.15	201.80133	172.97, 164.83, 157.06117.01
New-8	1.16	194.99686	177.03, 160.84, 129.01.75.00
New-9	1.18	336.06302	294.13, 253.11, 213.07, 188.08
New-10	1.20	449.02478	408.95, 301.51, 240.97, 152.91
New-11	1.20	365.03354	296.89, 210.95, 139.00, 129.01
New-12	1.22	547.08459	501.07, 339.01, 311.02, 255.06, 135.00
New-13	1.25	349.07632	179.05, 161.04, 129.01, 113.02
New-14	1.26	277.00293	258.99, 215.00, 197.04, 179.03, 135.04
New-15	1.27	266.97964	147.02, 129.01, 96.95
New-16	1.28	317.04922	197.00, 147.02, 129.01, 85.02
New-17	1.30	305.01953	244.96, 202.95, 133.01, 111.01
New-18	1.33	717.14008	669.81, 590.33, 365.07, 273.02, 175.02
New-19	1.35	541.10754	507.36, 450.26, 365.07, 291.00, 157.01
New-20	1.40	328.04636	301.03, 212.83, 152.88, 94.92
New-21	1.50	365.07339	296.89, 260.87, 210.95, 129.01
New-22	1.50	186.04048	142.05, 128.03, 115.01
New-23	1.56	277.0029	230.99, 215.00, 135.04, 79.95
New-24	1.73	365.07324	228.84, 175.02, 133.01, 113.02
New-25	1.86	203.05583	185.04, 159.06, 141.05, 113.05, 95.04
New-26	1.99	308.97482	229.01, 195.02, 151.03, 121.02, 96.95
New-27	4.14	315.07236	254.85, 219.84, 153.01, 109.02
New-28	5.02	258.99213	221.84, 215.00, 135.04, 79.95
New-29	5.51	439.03531	277.00, 258.99, 197.04, 179.03, 135.04
New-30	6.30	311.05728	282.87, 254.88, 240.04, 227.03
New-31	6.54	471.00742	229.01, 211.00, 179.03, 165.00, 135.04
New-32	6.84	439.03543	371.09, 302.84, 249.06, 215.00, 121.02
New-33	6.95	373.05643	255.03, 243.06, 223.07, 174.95, 146.96
New-34	7.35	439.03528	258.99, 215.00, 161.02, 135.04
New-35	7.63	417.08328	255.05, 211.06, 193.05, 179.03, 149.05
New-36	7.94	489.03574	397.02, 353.03, 341.03, 309.04, 280.99
New-37	8.13	162.83803	142.99, 127.86, 119.04, 103.91, 92.99
New-38	10.19	819.38354	351.05, 289.05, 193.03, 175.02, 113.02
New-39	12.10	485.32806	455.31, 409.11, 367.11, 340.98, 300.84
New-40	12.10	531.33356	485.32, 409.13, 310.87, 174.94, 136.30
New-41	12.48	487.34219	469.33, 443.35, 407.33, 322.99, 292.81
New-42	12.52	485.328	441.33, 408.57, 300.21, 258.54, 230.21
New-43	12.62	487.3421	487.34, 332.24, 300.54, 174.95, 146.95
New-44	13.05	311.22385	282.87, 254.88, 181.91, 171.10, 146.96
New-45	13.52	487.34219	441.33, 409.22, 300.89, 230.34, 174.95
New-46	14.64	469.3324	423.32, 409.31, 300.91, 265.89, 221.61, 191.94
New-47	14.74	471.34937	425.33, 300.92, 260.87, 229.86, 148.5, 102.39
New-48	14.92	471.3494	423.32, 299.89, 260.87, 184.42, 119.94
New-49	14.97	471.34692	423.32, 299.89, 260.87, 184.42, 119.94
New-50	15.28	601.37494	541.35, 479.35, 423.32, 300.84, 113.02
New-51	16.78	115.91959	99.92, 72.51, 66.07, 60.45, 51.89

### Analysis of Shuangxia Decoction Absorbed Into Blood Components With UPLC-QE-Orbitrap-MS

**FIGURE 2:**
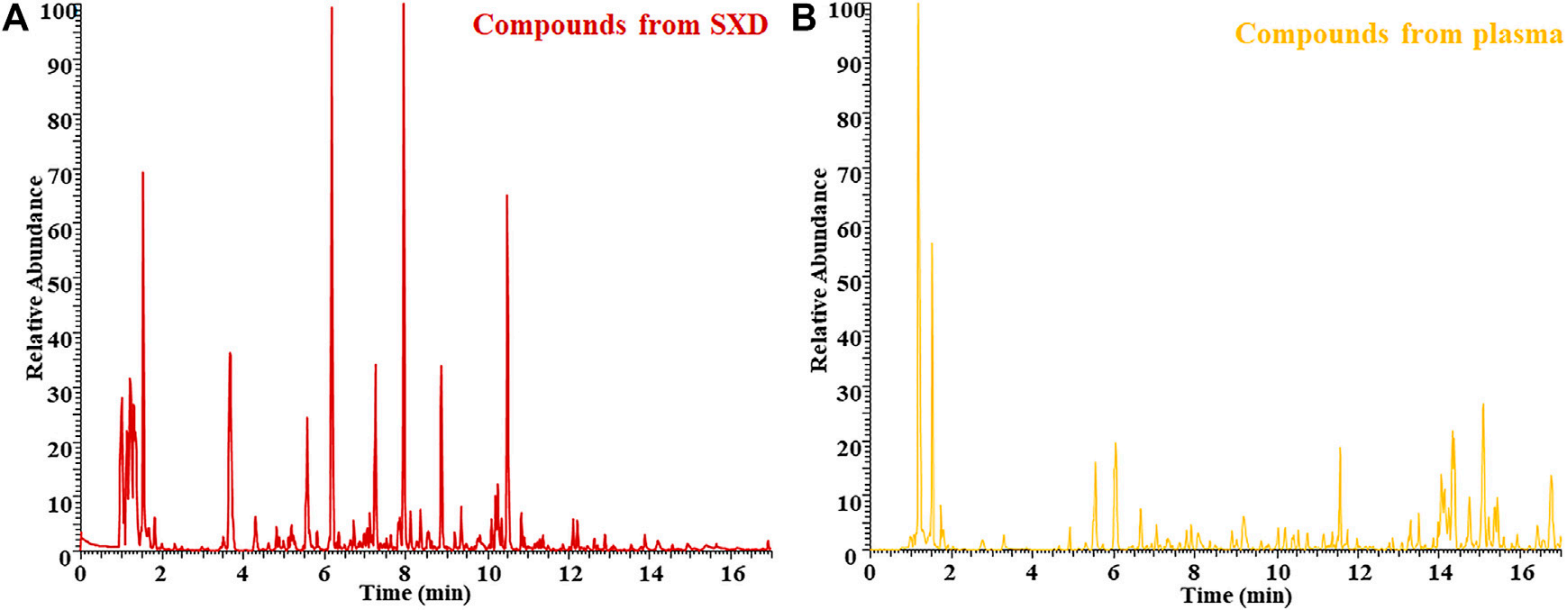
BPI chromatogram of Shuangxia decoction freeze-dried powder sample (SXD) **(A)** and the rat plasma sample after administration of Shuangxia decoction freeze-dried powder **(B)**.

Shuangxia decoction freeze-dried powder is dissolved in pure water, centrifuged, and analyzed by mass spectrometry. The negative ion mode BPI chromatogram is shown in Figure 2A, which was selected as the analysis object for the number of negative ion chromatographic peaks is far from the number of positive ion chromatographic peaks. Simultaneously, the drug-containing plasma of rats after one-week administration was analyzed by mass spectrometry, and the BPI chromatogram is shown in Figure 2B.

**FIGURE 3:**
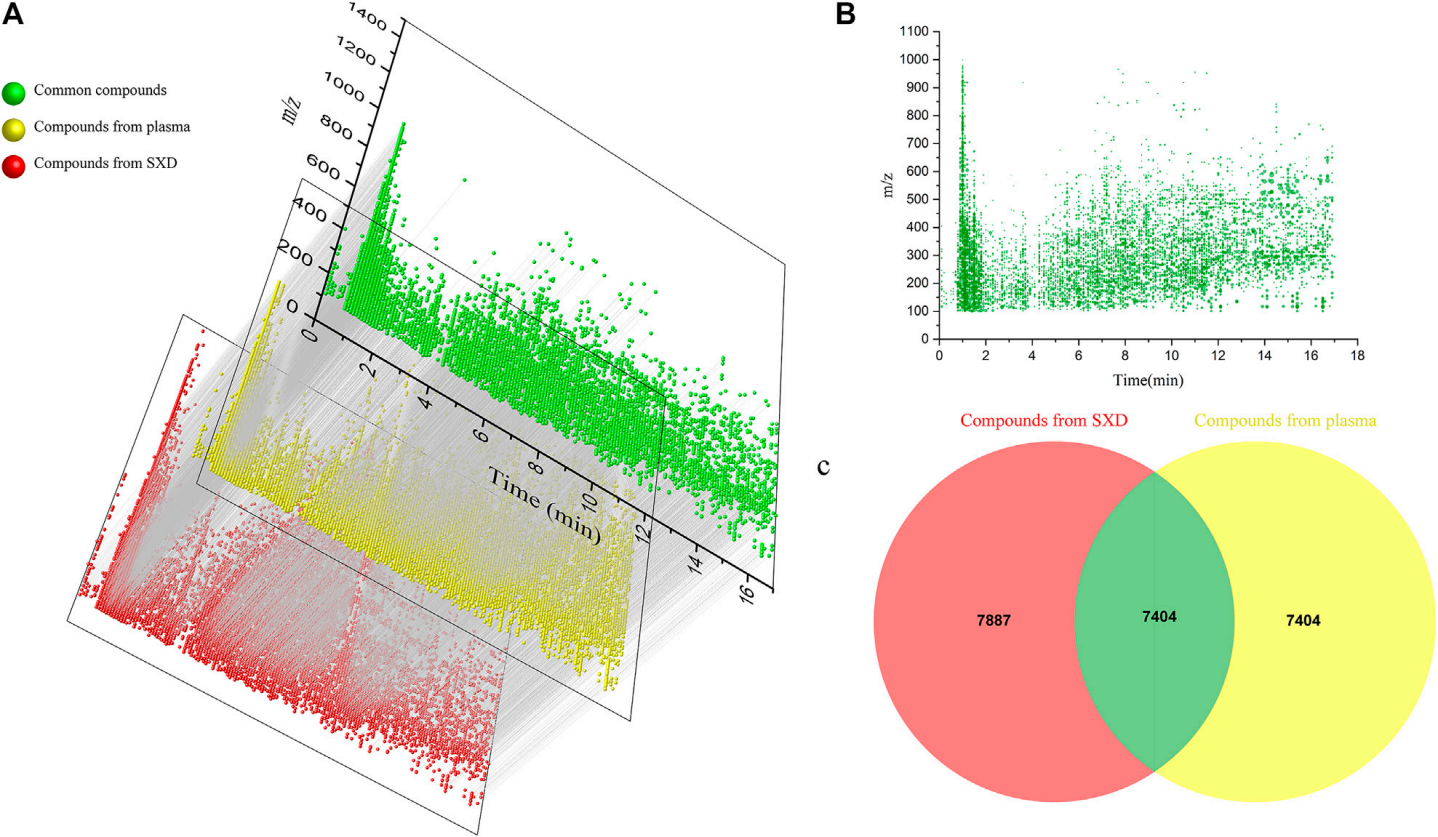
Mapping map of drug-derived component absorbed into blood from Shuangxia decoction. Red dots represent compounds in Shuangxia decoction, yellow dots represent all compounds in plasma after administration of Shuangxia decoction, and green dots represent compounds common to both samples **(A)**; distribution map of mass spectrum information of all absorbed blood components from Shuangxia decoction, the chromatographic retention time and m/z value of each compound are presented on the coordinate axis, and the relative normalized value is expressed by the size of the bubble **(B)**; the Venn diagram of all absorbed blood components. A total of 7,404 compounds were found to have been absorbed into the blood by mass spectrometry data intersection between the freeze-dried Shuangxia decoction and the plasma samples of rats treated with Shuangxia decoction **(C)**.

As described in 3.1, the raw mass spectrometry data of Shuangxia decoction extract and drug-containing plasma are normalized by MSDIAL by subtracting blanks and deleting the values below e7. Use retention time and m/z value to lock compounds, use Excel software to prepare bubble charts, and visualize all compounds, thereby reducing the invisible part of chromatographic peaks caused by differences in abundance. The origin software is used to map the compound of Shuangxia decoction extract and the compound in the plasma after administration, and the final common compound is all the compounds absorbed by Shuangxia decoction into the blood (Figure 3A). The mass spectrum information of the components from Shuangxia decoction absorbed into the blood is shown in Figure 3B, Venn diagram of the number of compounds contained in Shuangxia decoction extract and the number of compounds contained in plasma after administration Figure 3C. These figures objectively show all known and unknown components or metabolites absorbed into the blood.

**FIGURE 4:**
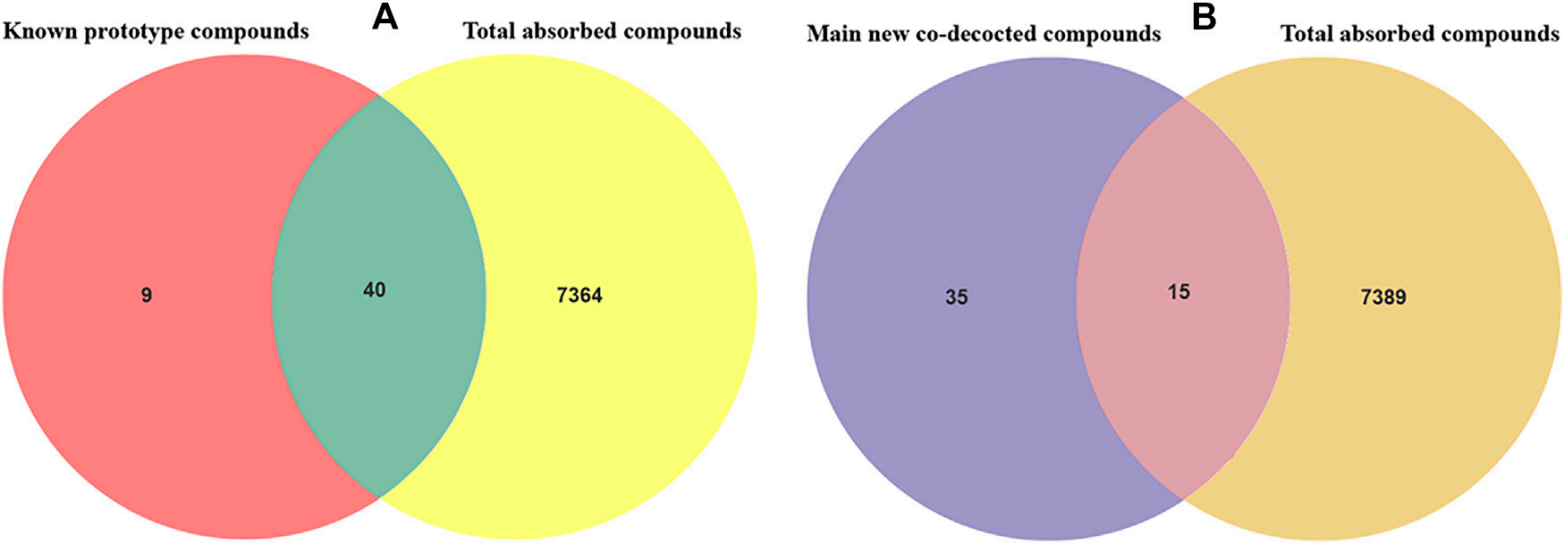
Venn diagram of the known prototype drug absorbed into the blood **(A)**; the Venn diagram of the main new generated compounds absorbed into the blood after co-decoction of Shuangxia decoction **(B)**. By the intersection analysis of the m/z values and retention time of the known prototype compounds and the m/z values and retention time of all the compounds absorbed into the blood from Shuangxia decoction, 40 known compounds were found to be absorbed into the blood. As indicated above, 15 new compounds were found to be absorbed into the blood after co-decoction.

By adopting the novel method of retention time vs m/z value to lock the compound, all the blood components after the administration of Shuangxia decoction are quickly screened, and the blood components and the identified components of the medicinal materials are again intersected as shown in Table 1, thereby derive the known prototypical composition into the blood. Forty known prototype components were definitely absorbed into the blood (Figure 4A), and 15 major new generated compounds were found to be absorbed into the blood (Figure 4B). The details are shown in Table 4.

**TABLE 4:** Known compounds absorbed into the blood from *Pinellia ternata (Thunb.) Makino*, *Prunella vulgaris* L. prototype ingredients, and the new generated ingredients by co-decoction.

Peak No	tR/min	Molecular Formula	MS/MS ions	Presumption
New-1	1.15	164.83542	136.93, 120.94, 108.93, 96.95	Codecoction
New-2	1.15	201.80133	172.97, 164.83, 157.06117.01	Codecoction
New-3	1.50	186.04048	142.05, 128.03, 115.01	Codecoction
P-4	1.55	C6H8O7	191.0191, 146.93767, 129.01826, 111.00761, 102.94759, 87.00752	Citric acid
P-5	3.52	C8H8O4	123.04395, 137.02327, 121.02818, 109.02829	Vanillic acid
P-6	3.69	C9H10O5	179.03409, 135.04399, 123.04397, 181.63736,	Danshensu
P-7	4.32	C7H6O4	153.01846, 142.94557, 114.95053, 109.02839	3,4-dihydroxybenzoic acid
P-8	4.63	C13H16O9	271.09769, 153.01830, 123.04393, 109.02822	5-(β-D-glucopyranosyloxy)-2-hydroxybenzoic acid
P-9	5.02	C11H12O7	211.06053, 193.04985, 149.05971, 137.05966	Piscidic acid
P-10	5.13	C7H8O2	95.04893, 93.03328, 81.03320	2,6-dimethyl-4H-pyran-4-one
P-11	5.29	C20H18O10	329.10272, 285.11304, 267.10257, 135.04404	Juglanin
P-12	5.59	C7H6O3	137.02328, 109.02833, 93.03327, 81.03313	Protocatechualdehyde
P-13	5.83	C17H16O8	303.08572, 285.07828, 259.09601, 215.10712	2.2′-[(6-oxo-7.8,9,10-tetrahydro-6h-benzo [c]chromene-1,3-diyl) bis(oxy)]diacetic acid
P-14	6.14	C9H6O4	135.04395, 133.02838, 105.03338	Esculetin
P-15	6.19	C9H8O4	179.07034, 151.07538, 135.04405, 103.91883	Caffeic acid
New-16	6.30	311.05728	282.87, 254.88, 240.04, 227.03	Co-decoction
P-17	6.66	C18H14O9	327.05099, 267.06601, 239.07123	2-(3,4-dihydroxyphenyl)-3,5-dihydroxy-7-methoxy-4-oxo-4H-chromen-8-yl acetate
P-18	6.73	C17H16O7	283.05994, 255.06682, 197.04469, 179.03406, 151.03899	2-(3,5-dimethoxyphenyl)-5,6,7-trihydroxy-2,3-dihydrochromen-4-one
P-19	6.82	C15H10O6	255.02980, 241.05019, 211.03934	Kaempferol
P-20	6.9	C15H10O4	237.05569, 209.06030, 181.06497	Daidzein
P-21	6.92	C27H30O16	511.08969, 271.02466, 301.03397, 151.00250	Rutin
New-22	6.95	373.05643	255.03, 243.06, 223.07, 174.95, 146.96	Co-decoction
P-23	6.99	C18H14O9	329.06647, 311.05618, 283.06094, 267.06613	3-(2,4-dimethoxyphenoxy)-5,7-dihydroxy-4-oxo-4H-chromene-2-carboxylic acid
P-24	7.06	C9H8O3	119.04900, 103.91891	p-coumaric acid
P-25	7.11	C21H20O12	301.03375, 271.02457, 255.02937, 243.02777	Hyperoside
P-26	7.13	C26H30O13	429.10483, 373.67609, 255.06601, 135.00764	Liguiritigenin-7-O-d-apiosyl-4′-O-D-glucoside
P-27	7.24	C21H22O9	343.08255, 255.06781, 147.02872, 135.04362	Liquiritin (Sh)
P-28	7.27	C24H26O13	521.13025, 359.10226, 329.13928, 197.04474, 179.03415, 161.02342	Salviaflaside
New-29	7.35	439.03528	258.99, 215.00, 161.02, 135.04	Co-decoction
New-30	7.63	417.08328	255.05, 211.06, 193.05, 179.03, 149.05	Co-decoction
P-31	7.83	C18H12O8	311.05591, 267.06598, 239.07066, 221.06064	2.2'-(1,8-dihydroxy-9,10-dioxo-9,10-dihydroanthracene-2,7-diyl) diacetic acid
P-32	7.87	C14H24O6	269.13937, 251.12854, 227.12833, 209.11768	(5-hydroxy-3-(hydroxymethyl)pentyl) hydrogen cyclohexane-1,2-dicarboxylate
P-33	7.96	C18H16O8	359.20828, 197.04474, 179.03416, 161.02341, 135.04396	Rosmarinic acid
P-34	7.96	C9H6O3	133.02832, 123.94485, 103.91898	7-hydroxycoumarin
P-35	8.14	C9H16O4	169.08612, 149.00063, 143.10666, 125.09598	Nonanedioic acid
P-36	8.28	C21H22O9	297.07666, 255.06596, 239.07158, 135.00763	Isoliquiritin
P-37	8.37	C27H20O12	355.04581, 311.05591, 267.06601, 239.07106	Sagecoumarin
P-38	8.58	C18H16O7	197.04475, 179.03413, 161.02341, 145.02841	Eupatorin
P-39	8.89	C15H12O4	255.06599, 153.01825, 135.0076, 119.049	Liquiritigenin
P-40	9.5	C26H20O10	311.05591, 267.06610, 239.07115, 135.04399	Salvianolic acid C
P-41	9.83	C18H32O5	291.19641, 229.14410, 211.13338, 171.10168	Seimatopolide A
P-42	10.13	C42H62O17	727.88745, 493.80930, 351.05685, 289.05618	Licoricesaponin G2
P-43	10.38	C15H12O4	255.06599, 153.01825, 135.0076, 119.04901	Isoliquiritigenin
P-44	10.52	C42H62O16	821.39679, 736.5127, 587.99011, 351.05655, 193.03452, 113.02319	Glycyrrhizic acid
P-45	10.86	C36H56O11	587.36243, 487.34280, 439.32211, 113.02319	Phytolaccoside B
P-46	11.39	C13H24O4	225.14903, 181.15903,	Tridecanedioic acid
P-47	12.12	C30H46O5	465.30151, 301.21738, 169.08649, 83.04895	Epiceanothic acid
New-48	12.48	487.34219	469.33, 443.35, 407.33, 322.99, 292.81	Co-decoction
New-49	12.52	485.328	441.33, 408.57, 300.21, 258.54, 230.21	Co-decoction
New-50	12.62	487.3421	487.34, 332.24, 300.54, 174.95, 146.95	Co-decoction
New-51	13.05	311.22385	282.87, 254.88, 181.91, 171.10, 146.96	Co-decoction
New-52	13.52	487.34219	441.33, 409.22, 300.89, 230.34, 174.95	Co-decoction
New-53	14.64	469.3324	423.32, 409.31, 300.91, 265.89, 221.61, 191.94	Co-decoction
New-54	14.74	471.34937	425.33, 300.92, 260.87, 229.86, 148.5, 102.39	Co-decoction
New-55	14.92	471.3494	423.32, 299.89, 260.87, 184.42, 119.94	Co-decoction

New, new generated ingredients by co-decoction; P, prototype ingredients.

### Confirm of Shuangxia Decoction Absorbed Into Plasma and Brain Prototype Components and Relative Content Analysis With UPLC-MS/MS

**FIGURE 5:**
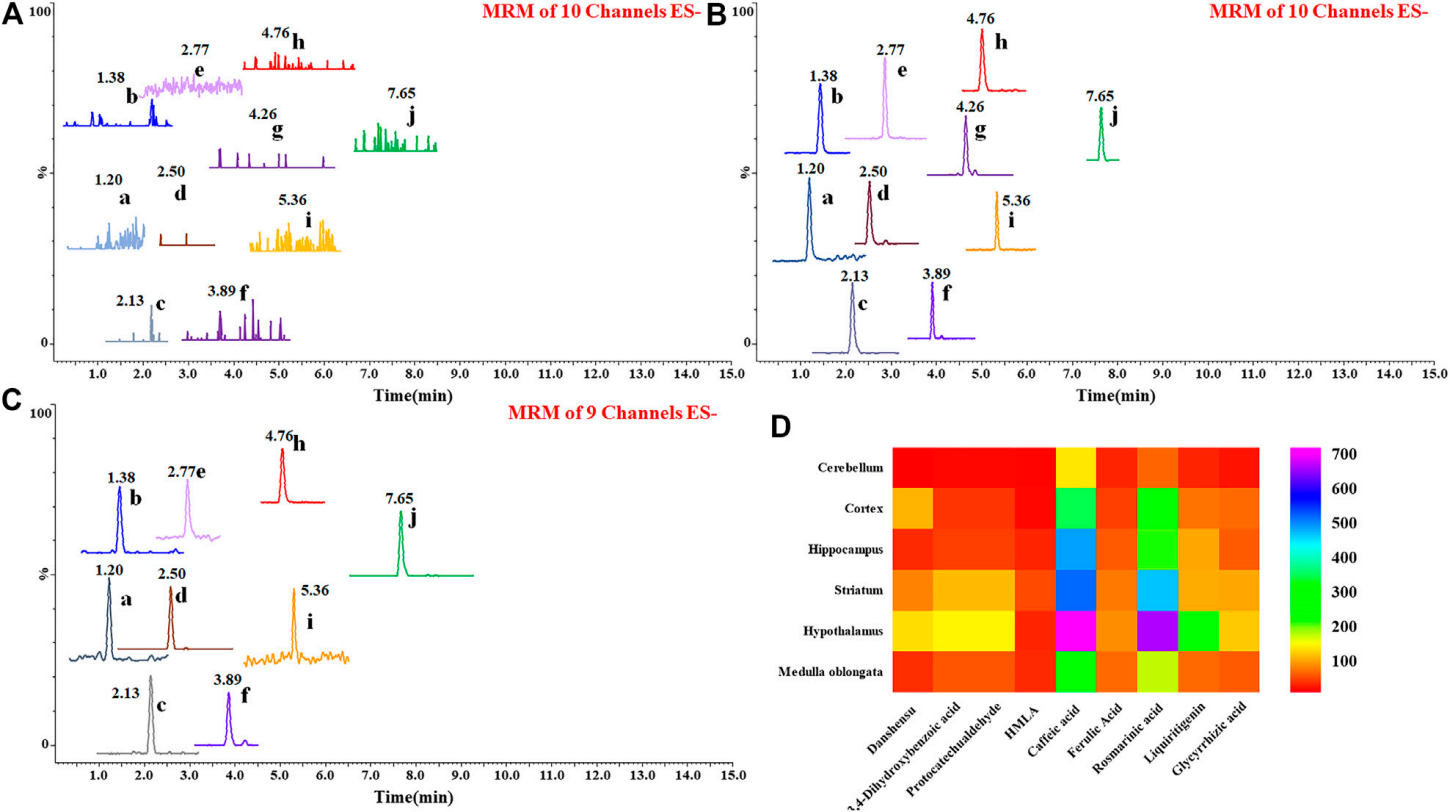
Chromatograms of 10 prototype compounds detected in blank plasma **(A)**, plasma **(B)**, and brain tissues after the last administration **(C)**. No endogenous interference was found in the blank matrix, 10 known compounds were accurately identified as being absorbed into the blood, and 9 compounds were accurately identified as being absorbed into the brain. 1) Danshensu, 2) 3,4-dihydroxybenzoic acid, 3) protocatechualdehyde, 4) HMLA, 5) caffeic acid, 6) ferulic acid, 7) salviaflaside, 8) rosmarinic acid, 9) liquiritigenin, and 10) glycyrrhizic acid. Heat map of relative content of 9 prototype drugs in Shuangxia decoction in different brain regions of rats **(D)**, and rosmarinic acid mainly accumulates in the hypothalamus, followed by the striatum. Caffeic acid is mainly distributed in the hypothalamus, striatum, and hippocampus.

After 7 consecutive days of administration, 1 h after the last administration, the rats were killed by anesthesia, and plasma was prepared by taking blood from the abdominal aorta, and then systemic perfusion, the hippocampus, striatum, hypothalamus, cortex, cerebellum, and medulla were stripped, wiped dry, and weighed. Add physiological saline in a weight-to-volume ratio of 1:3, use a vibration ball mill for rapid grinding, centrifuge and collect the supernatant, and process it according to the biological sample processing method. According to the experimental results (Figures 5A–D), it was found that after one week of administration, 10 compounds were confirmed in the plasma, which were derived from *Prunella vulgaris* L. and Glycyrrhizae Radix et Rhizoma (processing accessories). Furthermore, 9 compounds were confirmed in the hippocampus, cortex, hypothalamus, striatum, cerebellum, and medulla oblongata. These compounds all present a diffuse distribution, and there is no more obvious targeting in one nucleus among the brain. Even some ingredients from Glycyrrhizae Radix et Rhizoma extract can be detected in the brain.

### Molecular Docking Experiment Results

**FIGURE 6:**
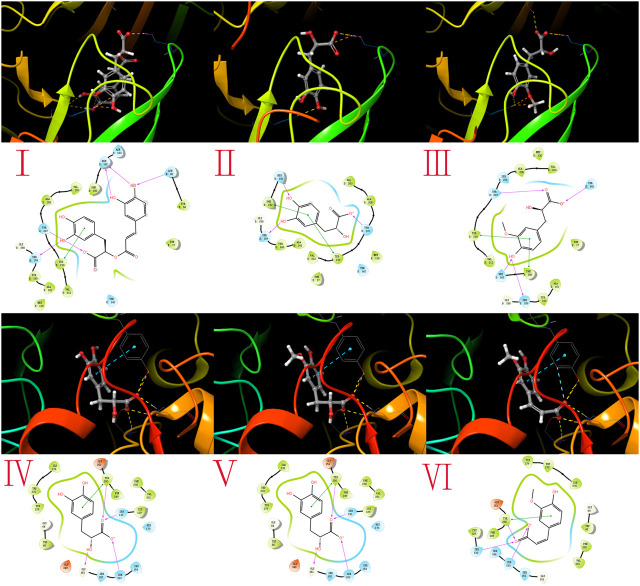
Molecular docking results. The action modes of potential active compounds with the classic sedative and hypnotic targets were calculated by Maestro 11.8, the 3D (upper) and 2D (lower) interaction diagram of GABA_A_ (6D6U), and dopamine D2 receptor (4MS4) with representative compounds are shown. **(A)** The action mode of rosmarinic acid and 6D6U; **(B)** the action mode of danshensu and 6D6U; **(C)** the action mode of HMLA and 6D6U; **(D)** the action mode of danshensu and 4MS4; **(E)** the action mode of HMLA and 4MS4; and **(F)** the action mode of ferulic acid and 4MS4.

The docking analysis and binding interactions were carried out using Maestro 11.8 software. As summarized in Table 5, the binding energies were computed to evaluate the binding affinities of the seven absorbed into the brain with three classic sedative and hypnotic neurotransmitter-related receptors GABA_A_ (6D6U), GABA_B_ (6LUQ), and dopamine D2 receptor (4MS4). It is believed that ligands owning more low binding free energies indicate more high binding affinities to their receptors. The binding free energies of the 7 compounds absorbed into the brain with the 3 receptors range from −3.1 to −10.5 kcal/mol, which indicated that these compounds may have potential sedative and hypnotic effects. The 3D and 2D action mode graphs of the representative compounds and related receptors are shown in Figure 6; among all ligands docking with 6D6U, rosmarinic acid exhibited the lowest energies (−10.5 kcal/mol), which is even lower than the theoretical value of the proligand. The action mode of rosmarinic acid with 6D6U was shown in Figure 6A, the phenolic hydroxyl of rosmarinic acid could form hydrogen bond with the active amino acid residue HIE102, ASN60, and SER159, the carboxyl could form a hydrogen bond with the active amino acid residue THR207, and the phenyl group could interact with TYR210 arene-H. Danshensu (Figure 6B) and HMLA (Figure 6C) had a similar binding mode.

**TABLE 5:** Molecular docking results of 7 prototype components absorbed into the brain from Shuangxia decoction.

Compound	6D6U	6LUQ	4MS4	Compound	6D6U	6LUQ	4MS4
Proligand	−8.6	−10.7	−11.4	Protocatechualdehyde	−8.2	−5.7	—
Rosmarinic acid	−10.5	—	—	Ferulic acid	−7.8	−5.3	−8.2
Danshensu	−9.8	−6.3	−9.5	Caffeic acid	−7.2	−6.4	—
HMLA	−8.7	−5.7	−9.5	3,4-dihydroxybenzoic acid	−3.1	−5.4	−7.4

Danshensu (Figure 6D) and HMLA (Figure 6E) also showed same better binding energy (−9.5 kal/mol) with 4MS4, which may be attributed to their extremely similar structure.

Ferulic acid also has a binding energy of −8.2 kal/mol, as shown in Figure 6F. Carboxylic and free hydroxyl of danshensu, HMLA, and ferulic acid could form hydrogen bond with the active amino acid residue GLY151, SER153, SER130, and TYR250.

## Discussion

At least for now, the mechanism of Chinese medicine compatibility has not been fully resolved, which has been plagued for the explanation of the mechanism of Chinese medicine and the development of modern Chinese medicine. According to the common sense of traditional Chinese medicine, the compatibility of Chinese medicine can reduce toxicity and increase efficiency. However, there is still no global analysis of how to reduce poison and how to increase efficiency. Different scientists try to explain the co-decoction of herbs and its final effect from different ideas. For example, some scientists have discovered that the coprecipitation produced by co-decocting traditional Chinese medicines may have special medicinal effects (Li et al., 2019). Some scientists have shown that co-decoction of different herbs may promote the dissolution of each other’s compounds or promote each other’s absorption, thereby improving the efficacy (Ma et al., 2019). Of course, some people speculate that the co-decocting of herbs may produce new compounds, but many times they remain in the imagination stage.

In previous studies, different scientists evaluated the sedative and hypnotic pharmacodynamics of the extracts of *Pinellia ternata (Thunb.) Makino* and *Prunella vulgaris* L., respectively. The results showed that the both herbal medicines own good sedative activity (Zhao et al., 2009; Wu et al., 2011). Our research team also assessed the pharmacodynamics of the formula of these two medicinal materials, namely, Shuangxia decoction, and the results proved that the curative effect was reliable (Pei et al., 2013; Xi et al., 2016; Zhang et al., 2016; Sun et al., 2020b). But so far, only few have comprehensively explored the sedative and hypnotic pharmacodynamic material from these two medicines and Shuangxia decoction *in vivo*. Only few literatures reported using the LC-MS/MS method to analyze the pharmacokinetic characteristics of few components from *Pinellia ternata (Thunb.) Makino* (Yang et al., 2018; Liang et al., 2020) nor have the direct effects of substances absorbed into the brain been reported from Shuangxia decoction. In this article, the ingredients from Shuangxia decoction that are absorbed into the blood are quickly identified, including 40 known prototype compounds and 15 co-decocted new compounds. These known compounds were derived from *Pinellia ternata (Thunb.) Makino*, *Prunella vulgaris* L., and their concocted accessory Glycyrrhizae Radix et Rhizoma. According to the theory of serum pharmacology, the compounds absorbed into the blood are the main contributors to the efficacy. Therefore, after the administration of Shuangxia decoction, not only the prototype compounds may be the effect contribution material but also the new generated compounds by co-decoction may be an effect substance. Therefore, the new substance generated by co-decocted also contribute to anti-diseases may be one of the traditional connotations of Chinese medicine compatibility.

Sleep-awakening is a complex neural activity that is easily disturbed by many factors, such as environment, foods, diseases, drugs, and emotions, and it is also regulated by a variety of neural structures in the brain in an orderly manner, including cortex, hypothalamus, locus coeruleus, basal forebrain, and midbrain reticular structure. There are a large number of nerve cells in the cerebral cortex, which dominate a variety of neural activities including sleep. The different phases of sleep are produced by the regulation of the cortex by multiple neural structures (hypothalamus and brainstem) under the cortex (Geng et al., 2015). According to our research results, rosmarinic acid is mainly distributed in the rat hypothalamus; therefore, it is very likely that rosmarinic acid acts on the ventrolateral preoptic area (VLPO) in the sleep- and wake-regulating nucleus. As one of the characteristic medicinal substances in *Prunella vulgaris* L and Shuangxia decoction, rosmarinic acid had been reported that it possesses sedative and hypnotic activity through activation of the GABA_A_-ergic systems within hypothalamic cells *in vitro* (Kwon et al., 2017). Therefore, our research results showed that rosmarinic acid is mainly distributed in the hypothalamus *in vivo*, which further confirms the reliability of the literature results. Simultaneously, rosmarinic acid is also found mainly in the striatum, which is closely related to Parkinson’s disease, so it is very likely that rosmarinic acid has a certain effect on the treatment of Parkinson’s disease. The caffeic acid is detected mainly in the hypothalamus and striatum in our research results, which may come from both Shuangxia decoction and the metabolism of rosmarinic acid (Baba et al., 2004). Literature reported that caffeic acid as a metabolite of chlorogenic acid can exert a mild arousal effect (Shinomiya et al., 2004). Therefore, caffeic acid may not be the active substance of Shuangxia decoction's sedative and hypnotic effect, but it may have the bidirectional regulation effect on the maintenance of sleep homeostasis and the effect of rosmarinic acid.

High-resolution mass spectrometry has extremely high resolution and sensitivity, which can detect substances with extremely different abundances, especially suitable for Chinese herbal medicine analysis. Although the efficacy of traditional Chinese medicine may be attributed to its main ingredients; however, the contribution of the trace components or new components produced after co-decoction to the efficacy of Chinese medicine is also worth paying attention. So far, the research on the contribution of the new ingredients after co-decoction to the efficacy of traditional Chinese medicine formulations is still blank. For a long time, ethnic medicine scientists tend to identify the main components of complex medicinal materials using high-resolution mass spectrometry, if we just stay here, everyone can use HPLC instruments to achieve their goals, or check the literatures to know the main medicinal substances contained in our common Chinese herbal medicines, after all, the HPLC instrument has already analyzed the main components of common Chinese medicines. Investigating trace components or new generated components may be more helpful to give full play to the functions of high-resolution mass spectrometry.

Although high-resolution mass spectrometry has high resolution and high sensitivity, it is difficult to confirm the structure of compounds, especially for new generated compounds. This also limits its wide application. After all, NMR is the gold standard for structural confirmation of organic compounds. However, NMR structural analysis requires the amount of the compound to reach the mg level, which is difficult to achieve for many newly formed compounds that are co-decocted. Therefore, the compound information that high-resolution mass spectrometry can provide is still worth having, after all, it lets us know that co-decoction does produce new substances. What is more, it lets us know that these new generated substances can also enter the blood and tissues. It allows us to re-understand more connotations of Chinese medicine compatibility. Therefore, we believe that our study is a small step forward in the re-understanding of the efficacy of Chinese medicines.

Traditional Chinese medicine administration usually takes longer, the target tissue generally has accumulation of medicinal substances, identifying the medicinal substances in the target tissue and exploring its mechanism of action can often achieve a multiplier effect with less effort. Mental diseases are not exactly the same as other tissue and organ diseases, because they often do not show some substantial lesions, which is often caused by some neurotransmitter disorders. Therefore, the interference of a series of factors related to the fate of neurotransmitters may cause brain diseases. It is described in the classic bible of traditional Chinese medicine that Shuangxia decoction can quickly exert a sedative and hypnotic effect when taken in large doses, which indicated that the effective substances in Shuangxia decoction may quickly enter the brain and cause central depression. Thus, it is more likely that the substances in Shuangxia decoction directly regulate the classic sedative and hypnotic targets. Therefore, exploring the components from Shuangxia decoction absorbed into the blood and absorbed into the brain, and then exploring its direct interaction with the powerful sedative and hypnotic receptors can explain the mechanism of Shuangxia decoction.

## Conclusion

For a long time, since the LC-MS technology and NMR technology cannot be perfectly combined, most of the people often carry out the next step of experimental research based on the known compounds confirmed by NMR, and many unknown compounds are often ignored, which is a discriminatory study caused by technical barriers. The co-decoction of traditional Chinese medicine formula may generate some new compounds, which may be contributors to the efficacy. This article uses mass spectrometry for the first time to explore the existence of these new generated compounds, known compound, and their absorption into the blood take Shuangxia decoction as an example. A novel data processing method to global analysis the display of the mass spectrum data of new compounds collected by LC-MS, and 51 new components produced by co-decoction have been discovered; furthermore, 15 of them were found to be absorbed into the blood. Forty of forty nine identified known compounds from Shuangxia decoction were quickly discovered to be absorbed into the blood. A total of 7404 compounds from Shuangxia decoction were found to be absorbed into the blood. Nine of the components absorbed into different brain regions are accurately identified and analyzed; rosmarinic acid, danshensu, and HMLA with GABA_A_ receptor have excellent binding characteristics. Danshensu and HMLA with dopamine D_2_ receptor also showed good binding energy. Our findings preliminarily reveal the components absorbed into the blood from Shuangxia decoction and its potential mechanism of action. The perfect combination of LC-MS and NMR still has a long way to go. Therefore, our method also provides confirmation and *in vivo* transfer of unknown new compounds after co-decoction, which may provide new ideas for further research on herbal .

## Data Availability

The original contributions presented in the study are included in the article/Supplementary Material, and further inquiries can be directed to the corresponding authors.

## Abbreviations

Co., Ltd., company limited; COVID-19, corona virus disease 2019; ESI, electrospray ionization; GABA, gamma-aminobutyric acid; HMLA, 4-hydroxy-3-methyloxyphenyl lactic acid; HPLC, high-performance liquid chromatography; 5-HT, 5-hydroxytryptamine; LC-MS, liquid chromatography-tandem mass spectroscopy; MRM, multiple reactions monitoring; MS, mass spectrometry; SPF, specefic pathogen free; UPLC-MS/MS, ultra high-performance liquid chromatography-tandem mass spectroscopy; UPLC-QE-Orbitrap-MS, ultra-high performance liquid chromatography-Q exactive-orbitrap-mass spectrometry; UPLC, ultra high-performance liquid chromatography
